# Supplementary material for: Molecular dynamics simulation based prediction of T-cell epitopes for the production of effector molecules for liver cancer immunotherapy
Source: PLoS One. 2025 Jan 3;20(1):e0309049. doi: 10.1371/journal.pone.0309049 (PMC11698456; doi:10.1371/journal.pone.0309049)
Supplement: S1 Table — (DOCX) [file pone.0309049.s001.docx]

**Supplementary Table 1**: List of bioinformatics tools/software used in this research study.

| **SOFTWARES** | **LINKS** | **USES** |
| --- | --- | --- |
| Human Protein Atlas | https://www.proteinatlas.org/ | Protein database |
| Liverome | http://liverome.kobic.re.kr/ | Protein database |
| VaxiJen | http://www.ddg-pharmfac.net/vaxijen/ | Antigenicity prediction |
| ExPasy- Compute pI/Mw tool | https://web.expasy.org/compute_pi/ | Molecular weight prediction |
| CELLO: Subcellular Localization Predictive System | http://cello.life.nctu.edu.tw/ | Subcellular localization prediction |
| HLAPred | http://crdd.osdd.net/raghava/hlapred/ | Epitopes prediction |
| UCSC Xena | https://xena.ucsc.edu/ | Conservation analysis |
| CamSol | http://www-vendruscolo.ch.cam.ac.uk/camsolmethod.html | Solubility prediction |
| ToxinPred | http://crdd.osdd.net/raghava/toxinpred/ | Toxicity prediction |
| ProtParam | https://web.expasy.org/protparam/ | Physiochemical properties prediction |
| GenomicScape-DNA Microarray | http://genomicscape.com/ | Expression sites analysis |
| IL-4Pred | http://crdd.osdd.net/raghava/il4pred/ | Interleukin 4 production |
| IL-6Pred | https://webs.iiitd.edu.in/raghava/il6pred/ | Interleukin 6 production |
| IL-10Pred | http://crdd.osdd.net/raghava/IL-10pred/ | Interleukin 10 production |
| IFNepitope | http://crdd.osdd.net/raghava/ifnepitope/ | Interferon gamma production |
| IEDB | https://www.iedb.org/ | Immunogenicity prediction, Proteasomal cleavage analysis |
| MOE | Installed Software | Binding affinity prediction |
| PEP-FOLD Peptide Structure Prediction Server - RPBS | https://bioserv.rpbs.univ-paris-diderot.fr/services/PEP-FOLD/ | 3D Modelling |
| TSNADB: Tumor Specific Neo-Antigen DataBase | http://biopharm.zju.edu.cn/tsnadb/browse/ | Tumor specific antigens prediction |
| TRON cell line portal | http://celllines.tron-mainz.de/ | Tumor associated antigens prediction |
| HAPPI | http://discovery.informatics.uab.edu/HAPPI/ | PPI |
| Cytoscape | https://cytoscape.org/ | PPI network |
| Robetta | https://robetta.bakerlab.org/ | 3D structures of proteins |
| ChimeraX | https://www.cgl.ucsf.edu/chimerax/ | Highlighting of epitopes in entire protein 3D structure |
| ERRAT | https://saves.mbi.ucla.edu/ | Quality analysis |
| C-IMMSIM | https://kraken.iac.rm.cnr.it/C-IMMSIM/index.php?page=1 | *In silico* cytokine release prediction |
| GROMACS | Online Software | Molecular Dynamics Simulation |
| Protein-sol Server | https://protein-sol.manchester.ac.uk/ | Solubility of polyvalent construct |
