# Supplementary material for: Molecular dynamics simulation based prediction of T-cell epitopes for the production of effector molecules for liver cancer immunotherapy
Source: PLoS One. 2025 Jan 3;20(1):e0309049. doi: 10.1371/journal.pone.0309049 (PMC11698456; doi:10.1371/journal.pone.0309049)
Supplement: S2 Table — (DOCX) [file pone.0309049.s002.docx]

**Supplementary Table 2:** Prediction, conservational analysis and population coverage of multiallelic MHC class-I T-cell epitopes.

| **Sr. No.** | **Protein** | **Epitopes** | **No. of alleles** | **Alleles** | **Region in Protein** | **SNP (%)** | **Population coverage** |
| --- | --- | --- | --- | --- | --- | --- | --- |
| 1 | AMBP | LGEGATEAE | 2 | HLA-A24, HLA-A*2402 | 70-78 | Nill | 21.38% |
| 2 | CDHR5 | EDIGTEADV | 6 | HLA-A*0201, HLA-A*0202, HLA-A24, HLA-A*3101, HLA-A*0301, HLA-A2.1, HLA-B14, HLA-B*5301 | 789-797 | Nill | 24.48% |
| 3 | CFB | LLYIGKDRK | 8 | HLA-B*3701, HLA-B40, HLA-B*4403, HLA-B*5301, HLA-B*51, HLA-B61 | 408-416 | Nill | 60.76% |
|  | VTN | QVDAAMAGR | 4 | HLA-A*3101, HLA-A*3302, HLA-A68.1, HLA-A20 | 345-353 | Nill | 20.53% |
| 5 | APOBR | HLEARKKSK | 9 | HLA-A2, HLA-A*0201, HLA-A*0202, HLA-A*0203, HLA-A*0301, HLA-A2.1, HLA-B*5301, HLA-B*0702, HLA-B*2704 | 135-143 | H135Y (68%) | 54.37% |
| 6 | AFP | HLCIRHEMT | 8 | HLA-A2, HLA-A*0201, HLA-A*0203, HLA-A*0206, HLA-A*0301, HLA-A2.1, HLA-B*5102, HLA-B*5103 | 483-491 | R487G (31%)  E489G (32%)  E489K (21%) | 55.02% |
| 7 | APOE | EQGRVRAAT | 7 | HLA-A2, HLA-A*0201, HLA-A*0202, HLA-A*0205, HLA-A20, HLA-A2.1, HLA-B*5301, HLA-B*51, HLA-B7, HLA-B*0702 | 204-212 | Nill | 62.81% |
| 8 | SERPINA1 | LKLSKAVHK | 10 | HLA-A2, HLA-A*0301, HLA-B27, HLA-B*2705, HLA-B62, HLA-B*0702, HLA-B*2704 | 351-359 | Nill | 55.45% |
