# Supplementary material for: Molecular dynamics simulation based prediction of T-cell epitopes for the production of effector molecules for liver cancer immunotherapy
Source: PLoS One. 2025 Jan 3;20(1):e0309049. doi: 10.1371/journal.pone.0309049 (PMC11698456; doi:10.1371/journal.pone.0309049)
Supplement: S3 Table — (DOCX) [file pone.0309049.s003.docx]

**Supplementary Table 3:** Prediction, conservational analysis and population coverage of multiallelic MHC class-II T-cell epitopes.

| **Sr. No.** | **Protein** | **Epitopes** | **No. of alleles** | **Alleles** | **Region in Protein** | **SNP (%)** | **Population coverage** |
| --- | --- | --- | --- | --- | --- | --- | --- |
| 1 | AMBP | VLGEGATEA | 3 | HLA-DRB1*0102, HLA-DRB1*0401, HLA-DRB1*0426 | 69-77 | V69M (23%) | 22.09% |
| 2 | CFB | WVTKQLNEI | 17 | HLA-DRB1*0701, HLA-DRB1*0703, HLA-DRB1*0801, HLA-DRB1*0802, HLA-DRB1*0804, HLA-DRB1*0813, HLA-DRB1*0817, HLA-DRB1*1102, HLA-DRB1*1114, HLA-DRB1*1120, HLA-DRB1*1121, HLA-DRB1*1301, HLA-DRB1*1302, HLA-DRB1*1322, HLA-DRB1*1323, HLA-DRB1*1327, HLA-DRB1*1328 | 334-342 | Nill | 37.79% |
| 3 | CDHR5 | VEEDTKVNS | 8 | HLA-DRB1*0301, HLA-DRB1*0305, HLA-DRB1*0306, HLA-DRB1*0307, HLA-DRB1*0308, HLA-DRB1*0309, HLA-DRB1*0311, HLA-DRB1*1107 | 133-141 | E135V (36%) | 18% |
| 4 | VTN | FTRINCQGK | 3 | HLA-DRB1*0408, HLA-DRB5*0101, HLA-DRB5*0105 | 210-218 | C215S (8%) | 42.27% |
| 5 | APOBR | WGILGREEA | 18 | HLA-DRB1*0101, HLA-DRB1*0305, HLA-DRB1*0306, HLA-DRB1*0307, HLA-DRB1*0308, HLA-DRB1*0309, HLA-DRB1*0311, HLA-DRB1*0802, HLA-DRB1*0813, HLA-DRB1*1101, HLA-DRB1*1114, HLA-DRB1*1120, HLA-DRB1*1128, HLA-DRB1*1302, HLA-DRB1*1305, HLA-DRB1*1307, HLA-DRB1*1321, HLA-DRB1*1323 | 275-283 | E281D (27%), E281K (20%) | 30% |
| 6 | AFP | LQDGEKIMS | 7 | HLA-DRB1*0301, HLA-DRB1*0305, HLA-DRB1*0306, HLA-DRB1*0307, HLA-DRB1*0308, HLA-DRB1*0311, HLA-DRB1*1107 | 278-286 | K283E (50%) | 18.92% |
| 7 | SERPINA1 | VKFNKPFVF | 14 | HLA-DRB1*0101, HLA-DRB1*0102, HLA-DRB1*0301, HLA-DRB1*0309, HLA-DRB1*1102, HLA-DRB1*1107, HLA-DRB1*1120, HLA-DRB1*1121, HLA-DRB1*1301, HLA-DRB1*1302, HLA-DRB1*1304, HLA-DRB1*1322, HLA-DRB1*1327, HLA-DRB1*1328 | 388-396 | Nill | 44.19% |
| 8 | APOE | VRAATVGSL | 12 | HLA-DRB1*0301, HLA-DRB1*0309, HLA-DRB1*0701, HLA-DRB1*0703, HLA-DRB1*0801, HLA-DRB1*0804, HLA-DRB1*0806, HLA-DRB1*1104, HLA-DRB1*1106, HLA-DRB1*1107, HLA-DRB1*1307, HLA-DRB1*1311 | 208-216 | Nill | 27.17% |
