# Supplementary figures and images for: Molecular dynamics simulation based prediction of T-cell epitopes for the production of effector molecules for liver cancer immunotherapy
Source: PLoS One. 2025 Jan 3;20(1):e0309049. doi: 10.1371/journal.pone.0309049 (PMC11698456; doi:10.1371/journal.pone.0309049)

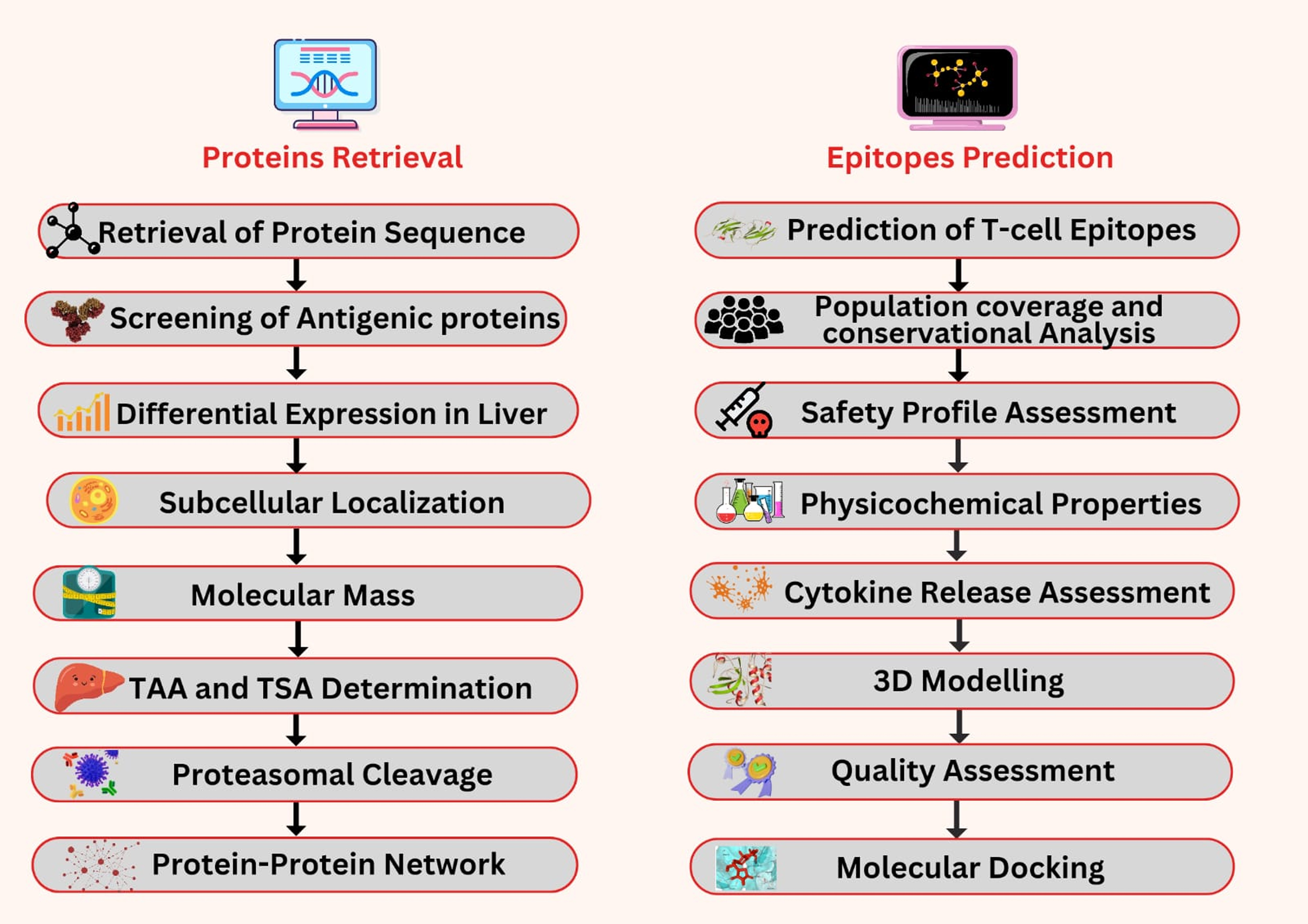

Supplement: S1 Graphical abstract — (TIF) [file pone.0309049.s004.tif]
