# Supplementary material for: Molecular dynamics simulation based prediction of T-cell epitopes for the production of effector molecules for liver cancer immunotherapy
Source: PLoS One. 2025 Jan 3;20(1):e0309049. doi: 10.1371/journal.pone.0309049 (PMC11698456; doi:10.1371/journal.pone.0309049)
Supplement: Attachment — Submitted filename: Response to Reviewers.docx [file pone.0309049.s005.docx]

**July 22, 2024**

**The Editor,**

PLOS ONE

**Subject: Re-Submission of the revised manuscript.**

**Manuscript No. ID. PONE-D-24-20487**

**Title: “Molecular Dynamics Simulation based Prediction of T-cell Epitopes for the Production of Effector Molecules for Liver cancer immunotherapy”**

We are thankful to the editorial board for useful comments and suggestions which helped us in improving our manuscript. Further suggestions would be appreciated if any.

We have modified the manuscript accordingly and individual responses to reviewer’s comments are listed below point by point:

**Report**

**Reviewer 1**

(1) In the materials and methods section, the authors should put the access link in front of the names of the software, servers, and databases.

**Author’s Response**

We are thankful for reviewer comments. As per recommendations of reviewer’s, Access links of all the software used in this research are added into the Supplementary Table 1 along with the names.

(2) The authors should explain why they set the antigenicity threshold to 0.5 in the manuscript. Do they have a reference for this value?

**Author’s Response**

We are thankful for reviewer comments. As per recommendations of reviewer’s, we have modified the manuscript and additional information have been highlighted in the manuscript at page no. 24 and line no. 543-545. Reference is added as per recommendations.

(3) Please calculate and report the aliphatic index and GRAVY parameters of the vaccine construct.

**Author’s Response**

We are thankful for reviewer comments. As per reviewer’s recommendations, we have revised the manuscript and changes have been highlighted in the manuscript at page no.19 and line no. 414 and 415.

(4) The authors should perform disulfide engineering of the vaccine construct.

**Author’s Response**

We are thankful for reviewer comments. As per recommendations of reviewer’s, we have performed the Disulfide engineering of our polyvalent construct and changes have been highlighted in the manuscript at page no. 9 and 20 at line no. 203-206 and 433-437 respectively. Figure 10 and Table 6 was added in result section.

(5) Please compare the characteristics of the vaccines designed in this study with the vaccines designed in other authors' studies. For this, please use the following references and cite them.

**Author’s Response**

We are thankful for reviewer comments. As per recommendations of reviewer’s, we have compared the characteristics of the provided paper links at page no.8 and 19 at line no. 175-177, 412 and 417 respectively also cited them in discussion portion.

**Reviewer 2**

(1) The manuscript is a bioinformatic analysis of TSA and TAA for Liver cancer. Although the immunoinformatic analysis, molecular dynamics analysis, immune simulation studies are also sufficient to predict the usage of this epitope-based vaccine but unless it is validated by using cancer cell lines or patients’ cells, the results cannot be confirmed. Animal model studies with immunogenic response and tumour regression is required to further validate their claim. So many such vaccine candidates are predicted for almost all the diseases and cancers specially in COVID times and are still being predicted, but they need to be validated preclinically or experimentally.

**Author’s Response**

We are thankful for reviewer comments. This is *in silico-*based research paper on designing and *in silico* screening analysis of peptides against liver cancer. These peptides are under experimental trials on rat models.

**Syed Aun Muhammad, PhD**

Corresponding Author
